# A somatic view of the genomic impact of mitochondrial endosymbiosis

**DOI:** 10.1371/journal.pbio.3002756

**Published:** 2024-08-23

**Authors:** Rose M. Doss, Martin W. Breuss

**Affiliations:** Department of Pediatrics, Section of Genetics and Metabolism, University of Colorado School of Medicine, Aurora, Colorado, United States of America

## Abstract

The endosymbiosis of mitochondrial ancestors resulted in the transfer of genetic material on an evolutionary scale for eukaryotic species. This Primer explores a new study in PLOS Biology which expands this to the genome of somatic cells within individuals and highlights its correlation with aging and disease.

## Teaser

The endosymbiosis of mitochondrial ancestors resulted in the transfer of genetic material on an evolutionary scale for eukaryotic species. A new study in *PLOS Biology* expands this to the genome of somatic cells within individuals and highlights its correlation with aging and disease.

The human genome is ever-changing—across generations but also within an individual. The latter drives a phenomenon called genomic mosaicism, where some but not all cells within a tissue harbor a unique “mosaic” mutation. The importance of this phenomenon has long been recognized in cancers; however, increasingly, it is appreciated as a driver of other diseases, a lineage mark for the otherwise experimentally intractable human development, or a recorder of mutational environments [1]. Almost any mutation type observed in the human germline can also be found as a mosaic event in a somatic cell or a collection thereof (i.e., as somatic mosaicism). However, their detection and proof of existence can be challenging. A new study by Zhou, Karan, and colleagues provides critical evidence that integrations of mitochondrial DNA into the human nuclear genome occur in typical somatic cells [2]. Moreover, their work suggests an intriguing correlation between this phenomenon and aging and disease.

The detection of genomic mosaicism is complicated by the need to access the specific tissue of interest, challenges when working with derived cells, and limitations of sequencing and analytical methodologies. Nevertheless, the field of genomic mosaicism has recently grown by leaps and bounds, mainly driven by technological advances; these now enable cost-effective deep next-generation sequencing, the amplification of single genomes, and the classification of mosaic variants through novel bioinformatic approaches from even “regular-depth” sequencing data [1,3]. This sparked a host of studies of non-cancer tissues, many of which focus on the human brain due to 2 main reasons: (1) brain somatic mosaicism holds the promise to unravel previously unsolved mysteries of human cognition and associated disorders; (2) neurons themselves represent a life-long postmitotic cell type, allowing an easy delineation of developmental and aging mosaicism [1,4]. While any mosaic variant may impact the function of a cell, large structural changes in the genome, which include the insertion of foreign or endogenous DNA elements, are more likely culprits than small ones.

One such potentially large germline variation is the integration of segments of mitochondrial DNA into the eukaryotic nuclear genome (“Numts”). This transfer of genetic information is generally thought to result from the endosymbiotic origin of mitochondria [5]. Indeed, “higher order” organisms harbor comparatively smaller mitochondrial genomes, suggesting an evolutionary transfer of information to the nucleus. While many Numts derive from such ancestral events, several studies also described their ongoing integration into the human genome [6,7]. Based on recent estimates, one in every 10,000 births harbors such an event de novo—i.e., present in the child but not either parent. While it is a rare occurrence relative to other types of variation (e.g., each newborn harbors dozens of novel single-nucleotide variants, and probably more than one in 50 carries a novel mobile element insertion [8,9]), this number pales in comparison to the tens of trillions of cells present in the human body. Thus, it is reasonable to surmise that somatic Numts exist—as also suggested by their presence in cancers. However, can we detect them? And do they actually matter?

**Fig 1:**
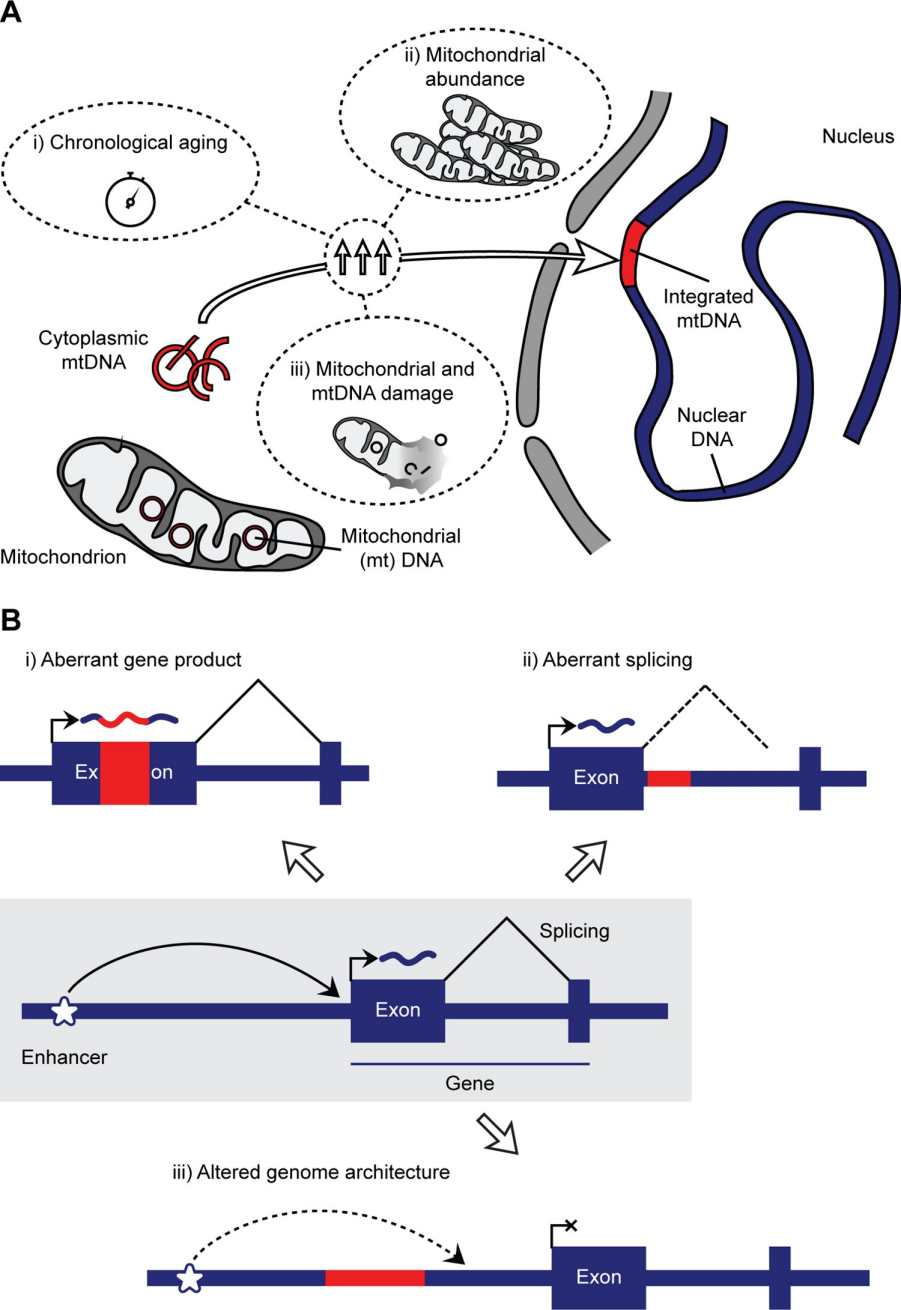
Origin and impact of mitochondrial DNA integration into the nucleus. (**A**) Mitochondrial DNA (mtDNA) can be released into the cytoplasm, transported into the nucleus, and integrated into the nuclear genome. This ongoing process can be observed across generations or within cancerous and non-cancerous somatic cells, and it is (i) driven by chronological aging, (ii) correlates with copy number of mitochondrial genomes, and (iii) accelerates in the presence of oxidative phosphorylation and mitochondrial and mtDNA damage. As the last 2 parameters can vary across tissues, between individuals, and in disease, the observed variable accumulation of Numts may be caused by them. Not shown is the potential impact of the import of mtDNA into the nucleus or its integration into the DNA itself, both of which have been established as modifiers of this process in yeast. Risk factors ii and iii likely act on the level of cytoplasmic mtDNA abundance; aging may impact all of the steps that are critical for numtogenesis. (**B**) Genome integrations, such as Numts, can impact the functional mammalian genome in several ways. Three potential outcomes of such an event are illustrated in simplified schematics: (i) disturbance of the coding sequence of a gene by integrating directly into an exon; (ii) interference with typical splicing; and (iii) alteration of the genome architecture (the example illustrates the loss of enhancer interaction and gene activation; however, other changes to, for instance, the 3D genome structure are possible). Outcomes i and ii may result in RNA degradation through nonsense-mediated decay or the expression of an altered protein product. Outcome iii may impact individual genes, groups of genes, or the overall genome integrity through a myriad of distinct mechanisms.

In this new study, the authors employed an adapted bioinformatic approach—based on their prior work—to answer these critical questions. Inspired by findings in yeast that suggested an association with aging, they assessed mosaic Numts in the ROSMAP cohort, which comprises hundreds of whole-genome sequencing datasets from older deceased individuals [10]. Critically, this also included data from various brain regions, as analyses focused on blood may miss somatic Numts due to their possible negative selection in bone marrow-derived cells. Together, this unique cohort enabled the authors to ask 2 fascinating questions: (1) Are there differences in somatic Numts across distinct brain regions and tissues? (2) How do aging and disease impact somatic “numtogenesis”? Surprisingly, the dorsolateral prefrontal cortex contained around 5 times more somatic Numts than the cerebellum, and their number is negatively associated with the age at death in individuals without cognitive impairment. Complementing their analysis of postmortem tissues, they drew on data from a previous in vitro “aging” experiment of fibroblasts that also included cells derived from an individual with *SURF1* mutations—associated with altered oxidative phosphorylation and mitochondrial DNA instability. Here, they found a chronological accumulation of somatic Numts, further exacerbated by impaired SURF1 protein function. Thus, the rate of numtogenesis is tissue specific and driven by chronological aging and mitochondrial DNA instability; the tissue specificity may simply be a product of mitochondrial number but may also be additionally fueled by differential mitochondrial function and damage (**Fig 1A**).

This study represents an important foray into somatic Numts in humans outside of cancers, provides evidence for their existence, and establishes their variability across tissues and with aging. While these variants generally increase with time, the authors observed an especially thought-provoking negative correlation with age at death. This finding suggests either a direct negative health impact of Numts or their correlation with likely mitochondrial risk factors that themselves cause earlier death. Despite this important caveat, it is easy to ascribe potentially harmful effects to the integration of Numts. Like viral or mobile element integration, these insertions of variable size may directly impact coding regions, patterns of splicing, or the overall genomic architecture (**Fig 1B**). However, the practical relevance of these mostly low-level somatic events remains unclear—a conundrum shared with many other mosaic variant types. Thus, whether they may be utilized as a correlative biomarker of pathogenic processes or are directly causative will have to be addressed in future studies.

What is next for somatic Numts? First, this important work will motivate the re-analysis of Numts across other available datasets to understand their presence, origin, and impact in more detail. Second, the ever-increasing application of third-generation long-read sequencing technologies and single-cell analyses in mosaicism studies will enable a more detailed understanding of this phenomenon. These next steps and exciting future discoveries are not unique to somatic Numts and shared with other mosaic variant types—they are, however, enabled by this present study that puts Numts into the spotlight of somatic mosaicism efforts.
